# MOFs-Derived Strategy and Ternary Alloys Regulation in Flower-Like Magnetic-Carbon Microspheres with Broadband Electromagnetic Wave Absorption

**DOI:** 10.1007/s40820-024-01416-2

**Published:** 2024-07-12

**Authors:** Mengqiu Huang, Bangxin Li, Yuetong Qian, Lei Wang, Huibin Zhang, Chendi Yang, Longjun Rao, Gang Zhou, Chongyun Liang, Renchao Che

**Affiliations:** 1 Laboratory of Advanced Materials, Shanghai Key Lab of Molecular Catalysis and Innovative Materials, Academy for Engineering & Technology, Fudan University, Shanghai, 200438 People’s Republic of China; 2 Department of Chemistry, Fudan University, Shanghai, 200438 People’s Republic of China; 3 Materials Genome Institute, Shanghai University, Shanghai, 200444 People’s Republic of China; 4 School of Materials Science and Engineering, Shanghai Institute of Technology, Shanghai, 201418 People’s Republic of China; 5 College of Physics, Donghua University, Shanghai, 201620 People’s Republic of China; 6 Zhejiang Laboratory, Hangzhou, 311100 People’s Republic of China

**Keywords:** Magnetic-carbon microspheres, MOFs derivatives, Electromagnetic wave absorption, Magnetic loss, Broadband absorption

## Abstract

Broadband electromagnetic (EM) wave absorption materials play an important role in military stealth and health protection. Herein, metal–organic frameworks (MOFs)-derived magnetic-carbon CoNiM@C (M = Cu, Zn, Fe, Mn) microspheres are fabricated, which exhibit flower-like nano–microstructure with tunable EM response capacity. Based on the MOFs-derived CoNi@C microsphere, the adjacent third element is introduced into magnetic CoNi alloy to enhance EM wave absorption performance. In term of broadband absorption, the order of efficient absorption bandwidth (EAB) value is Mn > Fe = Zn > Cu in the CoNiM@C microspheres. Therefore, MOFs-derived flower-like CoNiMn@C microspheres hold outstanding broadband absorption and the EAB can reach up to 5.8 GHz (covering 12.2–18 GHz at 2.0 mm thickness). Besides, off-axis electron holography and computational simulations are applied to elucidate the inherent dielectric dissipation and magnetic loss. Rich heterointerfaces in CoNiMn@C promote the aggregation of the negative/positive charges at the contacting region, forming interfacial polarization. The graphitized carbon layer catalyzed by the magnetic CoNiMn core offered the electron mobility path, boosting the conductive loss. Equally importantly, magnetic coupling is observed in the CoNiMn@C to strengthen the magnetic responding behaviors. This study provides a new guide to build broadband EM absorption by regulating the ternary magnetic alloy.

**Supplementary Information:**

The online version contains supplementary material available at 10.1007/s40820-024-01416-2.

## Highlights

- Metal–organic frameworks-derived CoNiM@C (M = Cu, Zn, Fe, Mn) microspheres were successfully fabricated with custom-built magnetic alloy-carbon heterogeneous interfaces.
- Flower-like CoNiMn@C microspheres achieve broadband electromagnetic wave absorption with effective absorption bandwidth of 5.8 GHz at only 2.0 mm thickness.
- Visual interface charge distribution and hierarchical magnetic coupling were observed to elucidate the electromagnetic energy absorption mechanism.

**Supplementary Information:**

The online version contains supplementary material available at 10.1007/s40820-024-01416-2.

## Graphical abstract

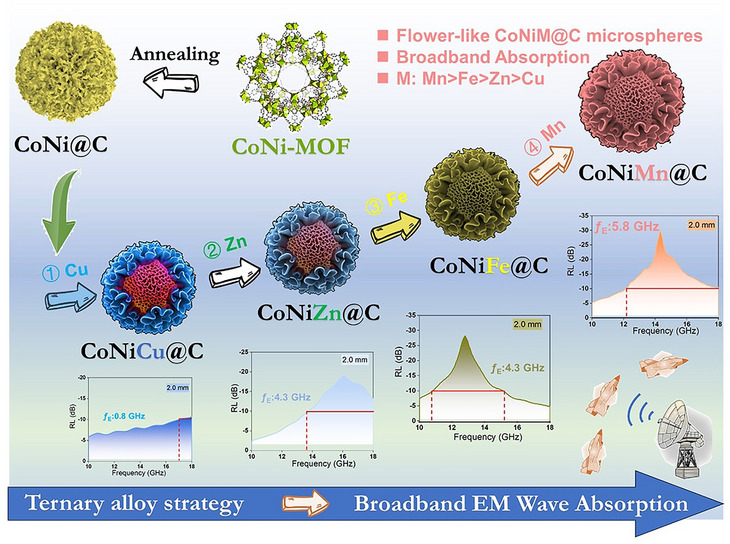

## Introduction

Communication technology, electromagnetic (EM) waves as carriers, has brought great convenience to people’s lives, but it brings EM radiation. Different with the EM interference shielding materials, the EM wave absorption materials can mainly convert EM energy into Joule energy, which is one of the effective means of purifying the EM environment [1–4]. Meanwhile, military radar stealth materials have higher requirements for EM wave absorption materials, including thin thickness, lightweight, broadband absorption, and strong absorption intensity [5–7]. Carbon materials have the advantages of wide sources, good corrosion resistance, and strong dielectric loss ability [8]. However, the single loss mechanism limits their further application. Therefore, introducing magnetic component into carbon materials not only optimize their impedance matching, but also provide magnetic loss toward incident EM wave.

As a classical magnetic alloy, cobalt–nickel (CoNi) alloy became the primary choice and search hotpot due to its intrinsic magnetic properties, responding ability, and absorption behaviors [9]. The regulation of microstructure and morphology is a strategy to tune their EM properties, minimum reflection loss (RL_min_), and efficient absorption bandwidth (EAB), such as nanoparticles [10], microflowers [11], microspheres [12, 13], and chain-like composites [9, 14, 15]. For example, Liu and co-workers studied the size-dominant absorption performance of CoNi microflowers, it was found that the strength of different stray magnetic fields contributed to the microwave absorption (RL_min_ =  − 25.8 dB, EAB =  ~ 5.4 GHz at 2.0 mm) [11]. Ma et al. found that the CoNi chains possess a stronger saturation magnetization and higher coercivity than CoNi particles because of their higher aspect ratios and larger crystalline sizes, exhibiting better absorption capacity (RL_min_ =  − 42.113 dB, EAB = 3.5 GHz at 5.4 mm) [9]. Gu et al. reported chain-like CoNi with enhanced magnetic loss, which contributed to the shape anisotropy and strong magnetic coupling effect, leading to the low frequency absorption (RL_min_ =  − 56.7 dB, EAB = 1.04 GHz at 4.1 mm) [15]. At the same time, many CoNi-based magnetic-carbon absorption system have been designed to maximize utilization of the magnetic-dielectric synergy effect, such as CoNi–CNTs [16], CoNi/C hybrids [17], CoNi@NC microspheres [18], CoNi@GC@C nanoboxes [19], and CoNi@MC composite [20]. The above results indicate that CoNi-based magnetic-dielectric absorption composites have great application potential.

Metal–organic frameworks (MOFs)-derived EM wave absorption materials attract considerable attention because of their diversity of structure and components, which can control the intrinsic EM properties and absorption properties [21–23]. MOFs-derived strategy provides huge design advantages to regulate structure and components: (i) the metal center can be transformed into different magnetic properties such as metal elements, oxides, and carbides; (ii) the similar morphology is basically maintained with adjustable specific surface area and pore size distribution; (iii) through post-processing, different atoms can be further doped to improve the conductivity of carbon components or change the local charge distribution. Based on the different organic linkers, bimetal MOFs precursors are obtained, including Co–Ni–BTC, MOF-74, ZIF-67 [24–26]. After post-processing, the bimetal host can be converted CoNi alloy, which further catalyzes graphitization transition of organic ligands. By controlling the periodic structure of ligands and the post-processing environment, various magnetic-carbon CoNi@C composites were fabricated toward EM wave absorption. For example, Liu e*t al.* synthesized bimetallic MOF-derived porous CoNi/C nanocomposites, combining advantages of excellent impedance matching and strong interfacial loss (RL_min_ =  − 74.7 dB, EAB < 4.0 GHz at 1.8 mm) [27]. Meng et al. constructed hollow CoNi/C composites stemmed from ZIF-67 as effective EM absorbers with an optimal RL_min_ value of − 61.8 dB at 3.9 mm and narrow absorption when the matching thickness is less than 2 mm [26]. Therefore, the strong EM wave absorption intensity can be achieved in CoNi/C composites. Combined with the material selection, the structural design in morphology regulation also displayed unique advantages in controlling nano–microstructure, components distribution, space charge distribution and tuning EM response capacity, which has been founded in reported literatures [28–30]. However, how to further expand the efficient absorption bandwidth is still facing a huge challenge.

Herein, ternary Co–Ni–M–MOFs-derived flower-like CoNiM@C (M = Cu, Zn, Fe, Mn) microspheres are prepared to construct the magnetic-dielectric synergy absorption materials. Compared with MOFs-derived CoNi@C microsphere, ternary CoNiM@C composites (CoNiCu@C, CoNiZn@C, CoNiFe@C, and CoNiMn@C) enhance the EM wave absorption performance after introducing the third element (Cu, Zn, Fe, Mn). Among those CoNiM@C microspheres, MOFs-derived flower-like CoNiMn@C microsphere hold the best broadband absorption and the efficient absorption bandwidth (EAB) can reach up to 5.8 GHz at 2.0 mm thickness, covering 12.2–18 GHz (~ 96.7% absorption of Ku band). The unique flower-like structure of the CoNiMn@C microspheres builds plentiful heterogeneous interface and hierarchical magnetic coupling, which therefore contribute harvest enhanced dielectric and magnetic loss. For the dielectric loss, the magnetic-carbon CoNiMn–C heterointerface encouraged the aggregation of the negative/positive charges to promote the interfacial polarization. And the graphitized carbon layer catalyzed by the magnetic CoNiMn core build the electronic mobility routes to boost conductive loss. Furthermore, hierarchical magnetic coupling is visually observed in the CoNiMn@C microspheres by off-axis electron holography technique, which benefits to magnetic loss capacity. In addition, micro-magnetic simulation and computer simulation technology (CST) simulation are characterized to explore the inherent absorption mechanism and application.

## Experiment Section

### Materials

CoCl_2_·6H_2_O, NiCl_2_·6H_2_O, FeCl_3_·6H_2_O, CuCl_2_, MnCl_2_·4H_2_O, ZnCl_2_, ethanol (C_2_H_5_OH, AR) and *N,N*-dimethylformamide (DMF, AR) got from Sinopharm Chemical Reagent Co., Ltd. 2,5-Dihydroxyterephthalic acid was purchased from Shanghai Aladdin Bio-Chem Technology Co., Ltd. All the chemicals and reagents were used without any further purification.

### Synthesis of CoNi@Carbon and CoNiM@Carbon Composites

Binary MOF precursors were prepared by a solvothermal process. Typically, CoCl_2_·6H_2_O (0.75 mol), NiCl_2_·6H_2_O and 2,5-dihydroxyterephthalic acid (0.48 g) were first dissolved in solution of DMF (60 mL), ethanol (3 mL) and water (3 mL). The mixed solution was stirred for 30 min and then was maintained at 160 °C for 6 h (100 mL autoclave). The products were obtained by washing (deionized water and ethanol several times) and drying under vacuum (60 °C for 12 h). Ternary MOF precursors were prepared by the same method except for the cations: CoCl_2_·6H_2_O (0.5 mol, 0.1188 g), NiCl_2_·6H_2_O (0.5 mol, 0.1190 g) and FeCl_3_·6H_2_O (0.5 mol, 0.1351 g) for CoNiFe-MOF, CoCl_2_·6H_2_O (0.5 mol, 0.1188 g), NiCl_2_·6H_2_O (0.5 mol, 0.1190 g) and CuCl_2_ (0.5 mol, 0.0672 g) for CoNiCu-MOF, CoCl_2_·6H_2_O (0.5 mol, 0.1188 g), NiCl_2_·6H_2_O (0.5 mol, 0.1190 g) and MnCl_2_·4H_2_O (0.5 mol, 0.0990 g) for CoNiMn-MOF, CoCl_2_·6H_2_O (0.5 mol, 0.1188 g), NiCl_2_·6H_2_O (0.5 mol, 0.1190 g) and ZnCl_2_ (0.5 mol, 0.0682 g) for CoNiZn-MOF, respectively. The products were annealed under a 5% H_2_ atmosphere in a tube furnace at 500 °C for 3 h for CoNiM@carbon composite.

### EM Wave Absorption Measurements

EM parameters of CoNiM@C samples were measured by vector network analyzer (VNA, a N5230C, at 2–18 GHz, 40 wt% in paraffin). According to the transmission line theory and the measured EM parameters, the RL value can be calculated to explore frequency-dependent performance. The formula is as follows [31, 32]:

1 $$ Z_{{{\text{in}}}} = \sqrt {\frac{{\mu_{r} }}{{\varepsilon_{r} }}} \tanh \left( {j\frac{2\pi fd}{c}\sqrt {\mu_{r} \varepsilon_{r} } } \right) $$

2 $$ {\text{RL}} = 20\log \left| {\frac{{Z_{{{\text{in}}}} - Z_{0} }}{{Z_{{{\text{in}}}} + Z_{0} }}} \right| $$

where Zin is the normalized input impedance, *ε*_*r*_ is the complex permittivity, *μr* is the complex permeability, *c* is the speed of light, *f* is the frequency, and *d* is the measured thickness.

### Characterization

The chemical composition, morphology, and microstructure of MOF-derived CoNiM@C composites were characterized by the X-ray diffraction (XRD, Bruker D8-Advance X-ray diffractometer), field-emission scanning electron microscopy (FESEM, S-4800), field-emission transmission electron microscopy (TEM, JEM-2100F), X-ray photoelectron spectroscopy (XPS, KRATOS Axis Ultra DLD), and superconducting quantum interference device magnetometer (MPMS VSM, Quantum Design Company).

## Results and Discussion

### Preparation and Characterization of the CoNiM@C Composites

**Fig. 1:**
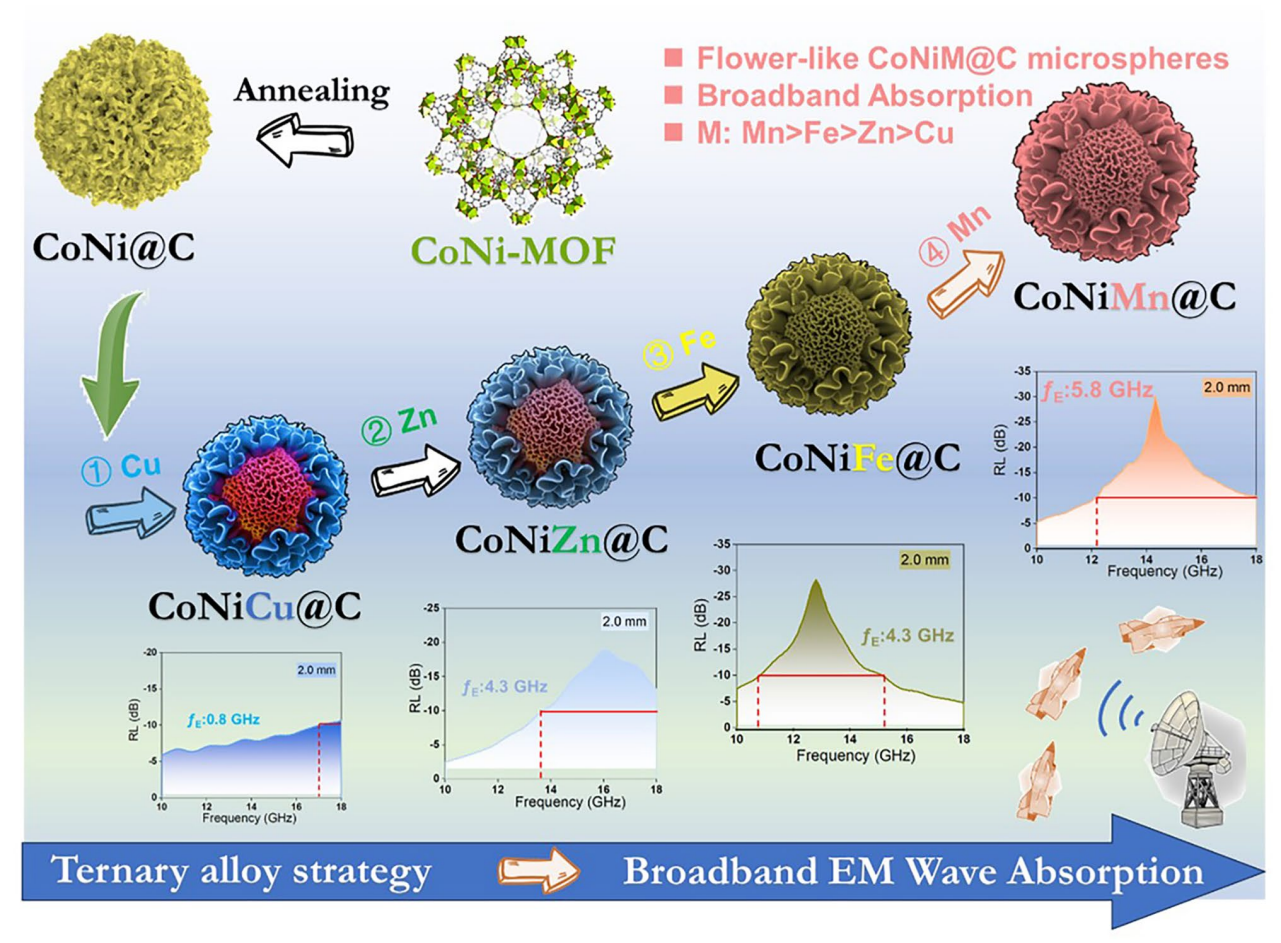
Design idea for broadband absorption in ternary MOFs-derived CoNiM@C microspheres

The synthesis process of MOFs-derived flower-like CoNiM@C microspheres is illustrated in Fig. 1. Using terephthalic acid as the organic ligand and the Co^2+^, Ni^2+^, and M^n+^ (Fe^3+^, Zn^2+^, Cu^2+^, Mn^2+^) as the metal main body, a series of ternary CoNiM-MOFs were prepared by a solvothermal method after heating at 160 °C for 6 h. After further calcined at 500 °C for 3 h at H_2_/Ar atmosphere, those metal main body transformed into metal alloy nanoparticles core high surface energy, which catalyzed the organic ligand into graphitized carbon shell. Finally, MOFs-derived flower-like CoNiM@C microspheres were successfully prepared with diverse metal components. After introducing the third metal elements into CoNi alloy, the intrinsic dielectric, magnetic, and electromagnetic properties can be adjusted to tune the broadband absorption.

**Fig. 2:**
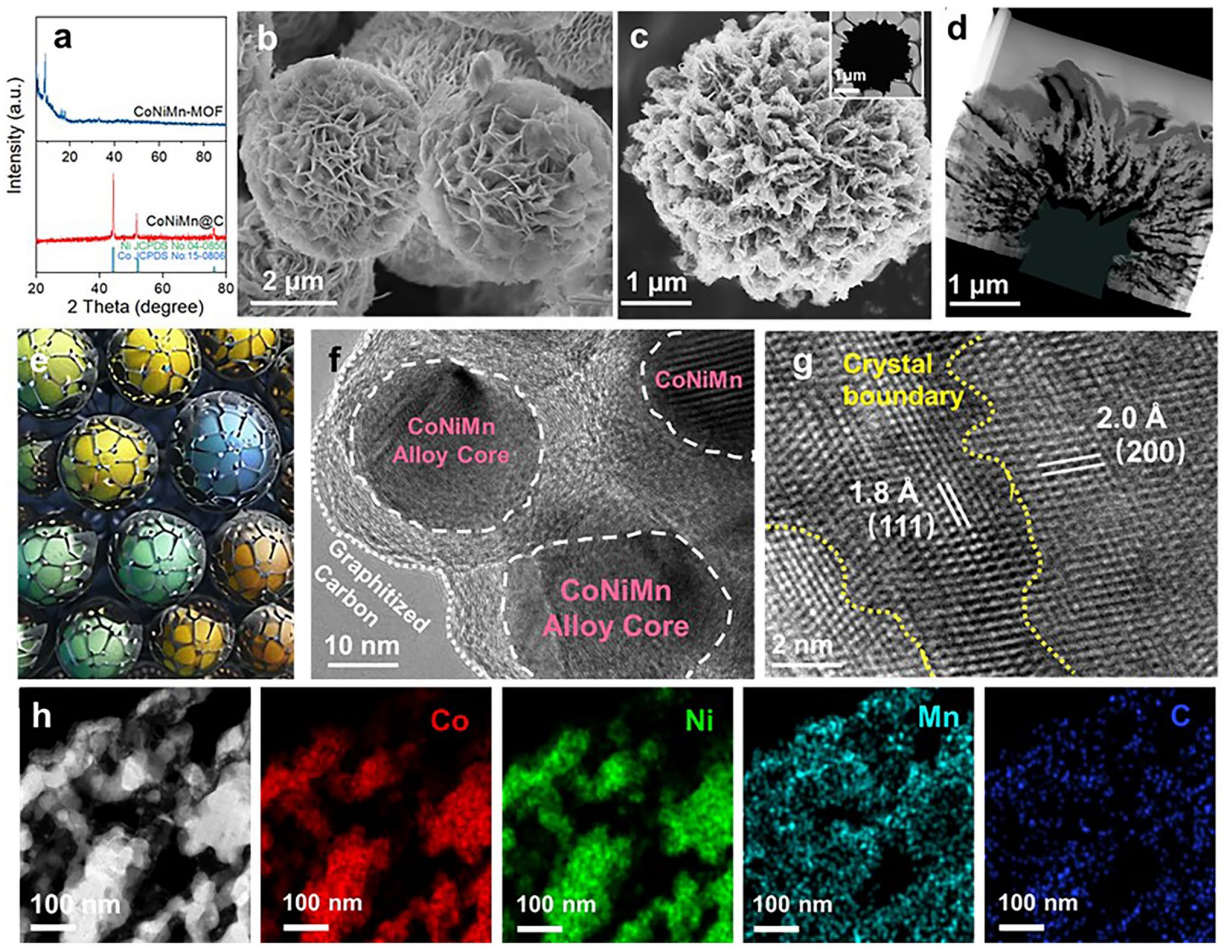
**a** XRD patterns of CoNiMn–MOF precursor and CoNiMn@C microsphere. SEM images of **b** CoNiMn–MOF and **c** CoNiMn@C. **d** TEM image via FIB slicing technique. **e** Diagram of alloy@C units. **f, g** HRTEM images. **h** HAADF image and corresponding element mapping

To validate the crystal structure and the purity of the obtained different flower-like microspheres, X-ray diffraction (XRD) measurements are conducted. In the XRD pattern, the precursors exhibit the standard characteristic peaks of MOFs. The diffraction peaks occurring at 2*θ* = 44.6°, 53.5°, and 74.0° belong to the *fcc* phase of the MOFs-derived alloy (Figs. 2a and S1) [33]. Subsequently, the morphologies and microstructures of the samples after annealing were recorded by SEM and TEM images. TEM image of pristine CoNiMn–MOF precursor is displayed in Fig. 2b. In Fig. 2c, the derived CoNiMn@C composite exhibits flower-like structure assembled by sheets with an average size of 4–5 μm. As expected, with the change of the third metal ion in the MOF precursor, the original morphology of CoNiM–MOF precursors was perfectly inherited through the carbonization process at 500 °C in H_2_/Ar stream because of its thermal stability (Fig. S2). The loosely stacked lamellar structure inside CoNiMn@C can be investigated by focus ion beam (FIB) slicing technique (Fig. 2d). What’s more, the elemental mapping that elements Co, Ni, Mn, Zn, Fe, and Cu are uniformly distributed in the flower-like microspheres, further confirming the successful preparation of CoNiM@C composites (Figs. 2h and S2). The flower-like microspheres are composed of CoNiMn alloy nanoparticles with a size of ~ 20 nm encapsulated by graphitized carbon (Fig. 2e, f). Moreover, many grain boundaries exist in the flower-like CoNiMn@C composite to form space charge regions, enhancing dielectric properties (Fig. 2g). XPS was performed to determine the chemical bonding states of flower-like CoNiM@C microspheres (Fig. S3). Two peaks located at 780.7 and 796.0 eV of the Co 2*p* are corresponded to the Co 2*p*_3/2_ and Co 2*p*_1/2_, respectively, and other two peaks at 787.3 and 803.8 eV belong to satellite peaks (Fig. S3a) [34]. The Ni 2*p* orbit consists of characteristic peaks at 853.7 and 872.2 eV, belonging to the binding energy of Ni 2*p*_3/2_ and Ni 2*p*_1/2_ (Fig. S3b) [35]. In the Mn 2*p* orbit, the peaks centered at 642.5 and 654.3 eV were ascribed to the binding energy of Mn 2*p*_1/2_ and Mn 2*p*_3/2_, which is consistent with the literature reports (Fig. S3c) [36]. XPS data of other samples also provided for complete comparisons (Fig. S3d–f).

### EM Wave Absorption Performance of the CoNiM@C Composites

Flower-like CoNiM@C material EM absorption performance depends on EM parameters composed by the complex permittivity (*ε*_*r*_ = *ε′ − jε′*′) and complex permeability (*μ*_*r*_ = *μ′ − jμ′′*). The real part (*ε*' and *μ*') of the complex permittivity and complex permeability indicate the storage capacity of EM energy [37, 38], while the imaginary part indicates the loss capacity of EM energy. The EM parameters of CoNiM@C composite were tested by vector network analyzer at the frequency of 2–18 GHz with 40 wt% composite content. And the covered frequency range (RL ≤  −10 dB) is defined as the effective absorption bandwidth [39].

**Fig. 3:**
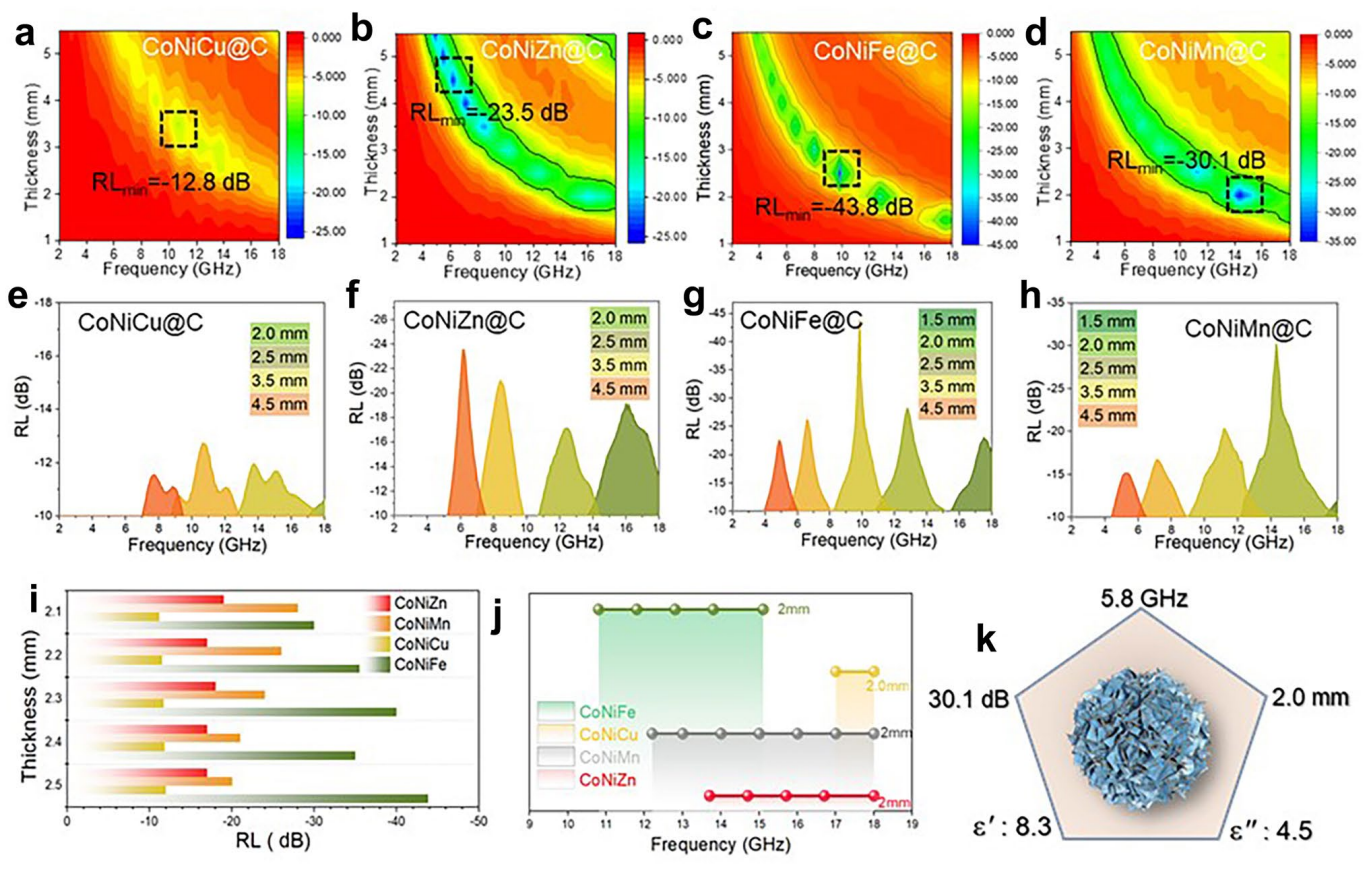
Reflection loss curves of **a, e** CoNiCu@C, **b, f** CoNiZn@C, **c, g** CoNiFe@C, **d, h** CoNiMn@C. The comparison of **i** reflection loss value with different thickness and **j** EAB for CoNiCu@C, CoNiZn@C, CoNiFe@C, and CoNiMn@C samples. **k** EM wave absorption performance of flower-like CoNiMn@C composites

As shown in Fig. 3, the ternary MOFs-derived CoNiM@C composites exhibit excellent EM absorption capacity and broadband absorption. The RL_min_ value of CoNiCu@C is − 12.8 dB at 10.8 GHz at 3.5 mm (Fig. 3a, e). The CoNiZn@C exhibits an RL_min_ of − 23.5 dB (6.1 GHz, 4.5 mm) and an EAB of 4.3 GHz at a thickness of 2.0 mm (Fig. 3b, f, j). The RL_min_ value of flower-like CoNiFe@C microsphere can reach as high as − 43.8 dB at 2.5 mm while the maximum EAB is 4.3 GHz at the same thickness (Fig. 3d, h, j). In the ternary MOF-derived CoNiM@C system, the CoNiMn@C microsphere with dielectric-magnetic synergy achieves both strong absorption capacity and wide absorption bandwidth. At a thickness of only 2.0 mm, the RL_min_ value of CoNiMn@C microsphere can reach − 30.1 dB at 14.2 GHz, and the maximum EAB can reach 5.8 GHz at only 2.0 mm (Fig. 3d, h, k). With thickness increased, the RL_min_ value of the CoNiMn@C system moves to the lower frequency region. By further comparing the properties of CoNiM@C, it is found that the reflection loss intensity is adjustable while CoNiFe@C exhibits the strongest EM wave absorption and CoNiMn@C achieves the widest EAB (Fig. 3i, j). Furthermore, the binary MOF-derived CoNi@C exhibits inferior magnetic characteristics than ternary CoNiFe@C because of the weak real and imaginary part of permeability and low saturated magnetization intensity (Figs. S4 and S5). Therefore, CoNiFe@C exhibits a stronger RL and a wider EAB when the sample thickness is 2.0 mm, which contributes to enhanced magnetic responding capacity (Figs. S6 and S7). Compared with other MOF-derived materials, the ternary MOFs derivatives achieve an excellent EM wave absorption performance, which provides new insights for the fabrication and design of broadband EM wave absorption materials (Table S1).

### EM Wave Absorption Mechanism of the CoNiM@C Composites

**Fig. 4:**
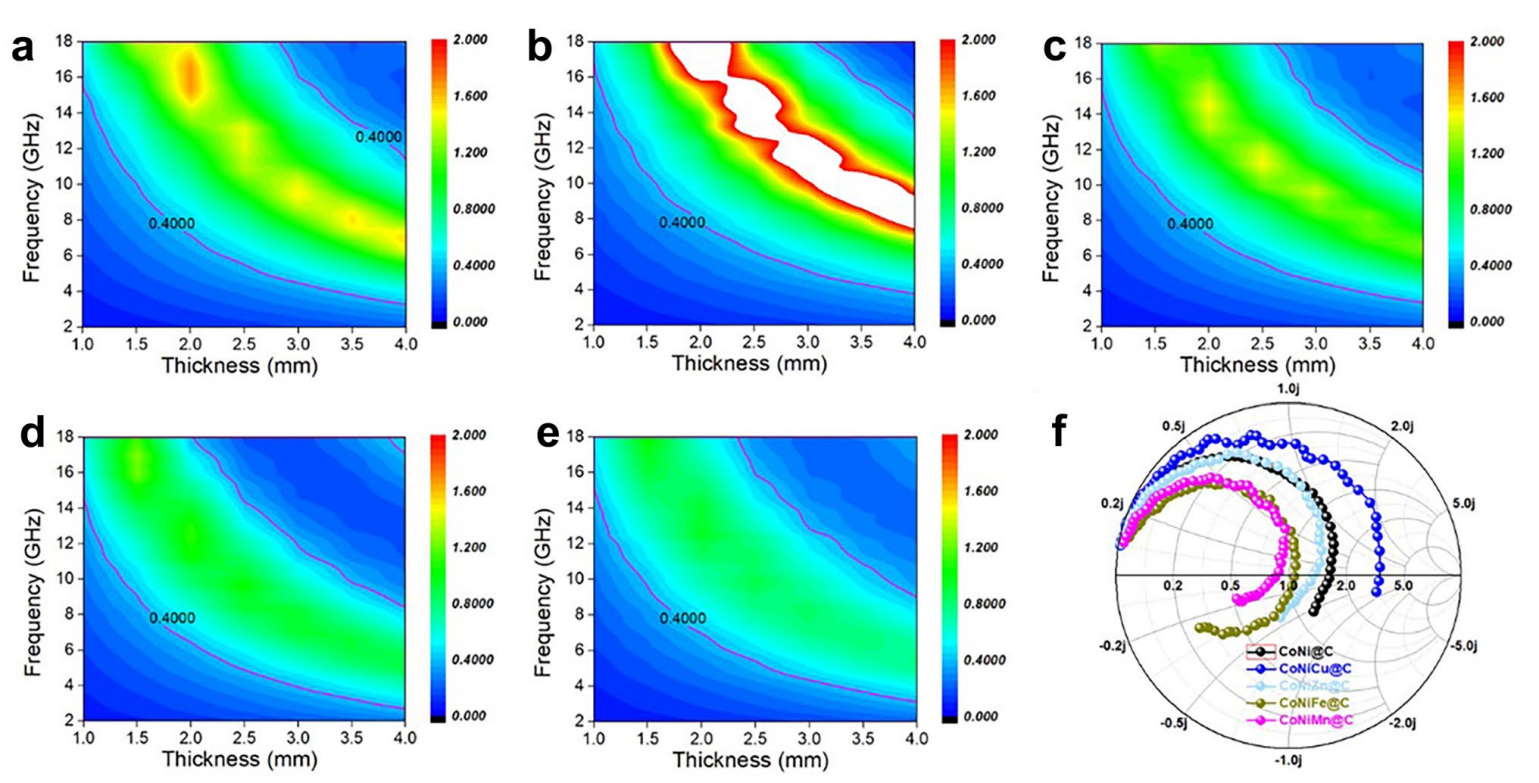
Impedance matching feature. The delta (*Δ*) function of **a** CoNi@C, **b** CoNiCu@C, **c** CoNiZn@C, **d** CoNiFe@C and **e** CoNiMn@C. **f** Smith chart of CoNiM@C composites when the thickness is 2.0 mm
  Furthermore, the Smith chart was further used to certify the impedance matching when the impedance thickness is 2.0 mm (Fig. 4f). In the Smith chart, the top part and bottom part belong to the inductive and capacitive regions, respectively [49]. For the Smith points, the closer to the center of Smith circle, the better the impedance matching is. And the shorter length means better impedance consistency over the frequency range. In Fig. 4f, the Smith circles of CoNiFe@C and CoNiMn@C composites are closer to the center, revealing the better impedance facing incident EM wave. The results elucidate that inducing adjacent elements into the magnetic CoNi core can regulate the EM responding behaviors and further boost the impedance matching.
(ii) **Tuning ternary magnetic core to enhance alloy-carbon interfacial polarization and conductive loss**. As an important component of dielectric absorption, polarization loss and conductive loss both contribute to high-performance EM energy dissipation, which is reflected by their EM parameters. The complex permittivity of MOFs-derived CoNiM@C microspheres is discussed to analyze their intrinsic dielectric performance. Based on the CoNi@C composite, adjacent elements from Periodic Table of Elements are induced into the inner magnetic core, respectively, forming different ternary alloy (Fig. 5a). Due to the changed magnetic components, constructed magnetic-carbon heterointerfaces and graphitized carbon layers will lead to different EM properties and complex permittivity.

Through element introducing and MOFs-derived strategy, ternary MOFs-derived CoNiM@C composites with flower-like nano–microstructure exhibited outstanding EM loss ability and broadband absorption performance. To better understand the energy dissipation mechanism, related magnetic–dielectric features have been explained in detail as follows:

(i) **Ternary magnetic core strategy to regulate the EM responding behaviors and impedance matching**. Theoretically, the outstanding EM wave absorption performance mainly contributes to well impedance matching and strong absorption together, which the former is primary [40–42]. Therefore, the incident EM waves should enter the interior of the CoNiM@C absorbers as much as possible, rather than reflect on its surface to meet the impedance matching requirement. Then, electric dissipation can be divided into two types including conductive loss and dielectric loss, which converts EM energy into thermal energy [43, 44]. Magnetic loss can consume EM energy through natural resonance (< 8 GHz) and ferromagnetic resonance (8–18 GHz) [45]. When the alternative high-frequency EM wave reaches the composites surface, ideal impedance matching commands that the impedance of CoNiM@C is equal to the impedance of the free space. To assess the impedance matching feature, the delta function (Δ) of CoNi@C and CoNiM@C microspheres were calculated by the following formulas [46, 47]:
  3 $$ \left| \Delta \right| = \left| {\sinh^{2} \left( {Kfd} \right) - M} \right| $$
  4 $$ K = \frac{{4\pi \sqrt {\mu^{\prime}\varepsilon^{\prime}} \times \sin \left( {\frac{{\delta_{e} + \delta_{m} }}{2}} \right)}}{{c \times \cos \delta_{e} \times \cos \delta_{m} }} $$
  5 $$ M = \frac{{4\mu^{\prime}\cos \delta_{e} \times \varepsilon^{\prime}\cos \delta_{m} }}{{\left( {\mu^{\prime}\cos \delta_{e} - \varepsilon^{\prime}\cos \delta_{m} } \right)^{2} + \left[ {\tan \left( {\frac{{\delta_{e} - \delta_{m} }}{2}} \right)} \right]^{2} \times \left( {\mu^{\prime}\cos \delta_{e} + \varepsilon^{\prime}\cos \delta_{m} } \right)^{2} }} $$
  Generally, well impedance matching holds a small |*Δ*| value, where the |*Δ*| is lower than 0.4 and with a larger area [48]. In Fig. 4a–e, the impedance matching feature is displayed. For the CoNi@C microspheres, it holds low |*Δ*| values at large area, which dominates well impedance matching (Fig. 4a). After inducing third element into the magnetic alloy, related impedance matching ability show changes. All the CoNiM@C microspheres exhibit well impedance matching except for the CoNiCu@C composite (Fig. 4b–e). And the order of general impedance matching ability is CoNiMn@C > CoNiFe@C > CoNiZn@C > CoNi@C > CoNiCu@C. Compared with other MOFs-derived composite, flower-like CoNiMn@C display the well matching behaviors at broadband frequency region, which is highly correlated with its intrinsic EM properties. Therefore, incident EM wave easily enters the CoNiMn@C composites when it propagates to the surface of materials. At the same time, CoNiMn@C hold the huge potential to obtain wide bandwidth absorption because of broadband responding capacity and matching behaviors.

**Fig. 5:**
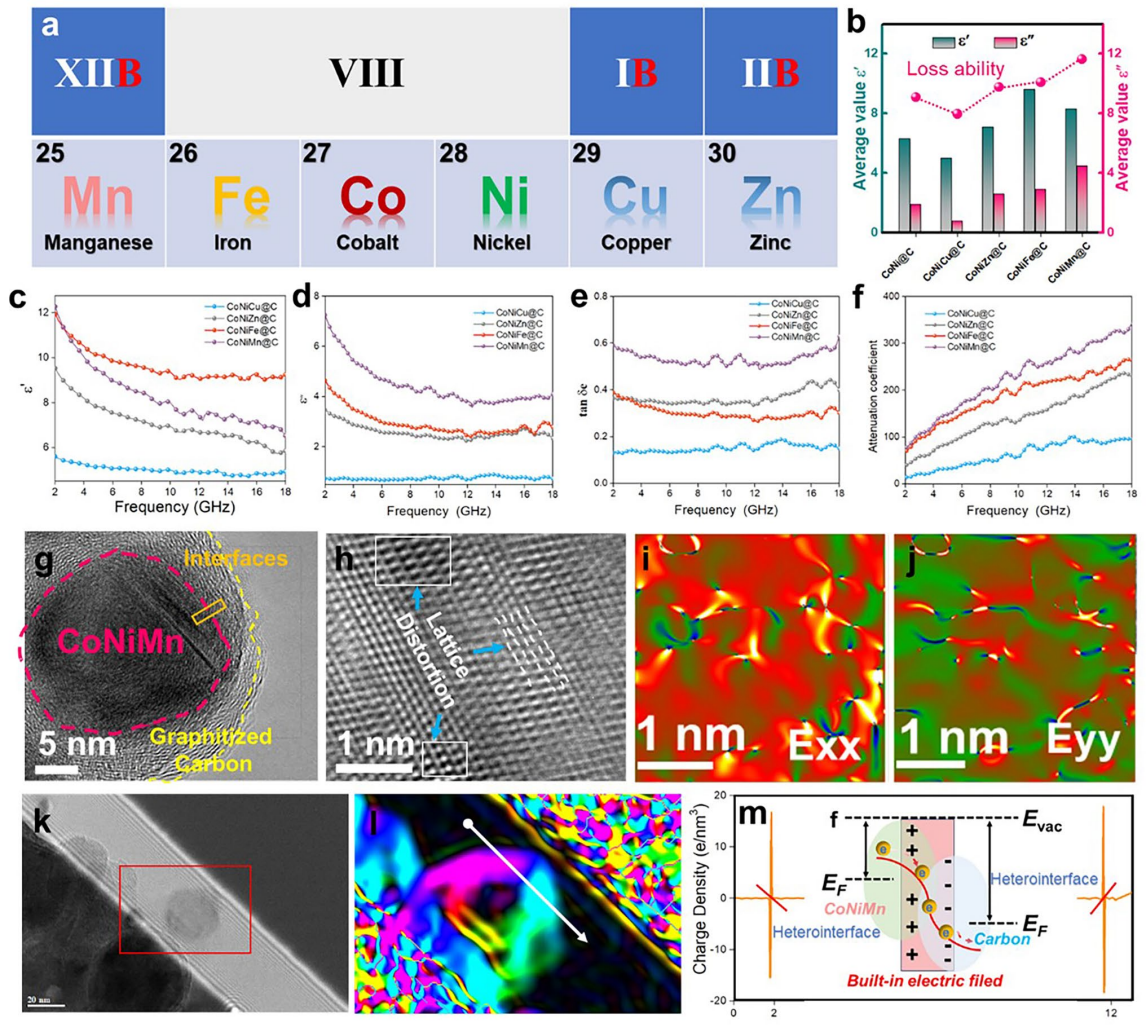
**a** Location of adjacent introducing element in periodic table of the elements. **b** Average ε′ and ε′′ value of CoNi@C and CoNiM@C. **c** Real and **d** imaginary part of permittivity, **e** tan δ_e_, and **f** attenuation constant of CoNiM@C microspheres. **g, h** HRTEM image and **i, j** strain distribution images along the E_xx_ and E_yy_ directions. **k** Holography image, **l** reconstructed phase image and **m** profile of charge density along the direction of white arrow in **i**
  As displayed in Fig. 5c, d, both the *ε'* and *ε"* curves of MOFs-derived CoNiM@C composites display the frequency-dependence feature. As measured frequency increased from 2 to 18 GHz, the *ε'* values were decreased from 5.6 to 4.9, from 9.5 to 5.9, from 11.9 to 9.2, and from 12.3 to 6.5, corresponding to the CoNiCu@C, CoNiZn@C, CoNiFe@C, and CoNiMn@C, respectively (Fig. 5c). Similarly, the *ε"* values were decreased from 0.75 to 0.73, from 3.5 to 2.4, from 4.6 to 2.8, and from 7.3 to 4.0 for CoNiCu@C, CoNiZn@C, CoNiFe@C, and CoNiMn@C, respectively (Fig. 5d). It can be found that after inducing the adjacent element (Zn, Fe, and Mn), the average *ε'* values increased from 6.3 of CoNi@C to 7.1 of CoNiZn@C, 9.6 of CoNiFe@C, and 8.3 of CoNiMn@C, respectively (Fig. 5b). For the *ε"* values, it was also displayed an increasing tendency, which rising from 1.9 of CoNi@C to 2.6, 2.9, and 4.5 for CoNiZn@C, CoNiFe@C, and CoNiMn@C, respectively (Fig. 5b). As we know, the imaginary part (*ε"*) of complex permittivity reflected the dielectric loss ability, which is highly related to intrinsic electronic conductivity (*σ*), heterointerface and defects. In those annealing process, metal cations converted into the metal alloy in the reducing H_2_/Ar atmosphere. Meanwhile, the metal alloy nanoparticles with high chemical activity can be acted as the catalyzer to catalyze the graphitization transformation of the organic linkers. Therefore, the catalytic capacity of reduced metal alloy plays the key role in dominating the electronic migration ability. Due to the strong catalytic capacity from the CoNiFe and CoNiMn nanoparticles, combined with the heterointerface and defects, the complex permittivity of MOFs-derived CoNiFe@C and CoNiMn@C displays the dielectric loss potential. On the other hand, loss angle tangent value (tan *δ*_e_) and attenuation coefficient (*α*) also demonstrate that the MOFs-derived CoNiMn@C possess the highest dielectric loss ability (Fig. 5e, f). Taking CoNiMn@C as an example, related dielectric loss is discussed in detail to understand the energy conversion process. As shown in Fig. 5g, the magnetic CoNiMn core was wrapped by the graphitized carbon layers, building the nanoscale CoNiMn–C heterointerface. The CoNiMn@C microsphere is assembled by plenty of nano CoNiMn@C units to construct high-density magnetic-carbon heterointerfaces. By the off-axis electron holography technology, the charge distribution can be obtained based on the reconstructed phase image, where originate from corresponding holography image (Fig. 5k, l). In the charge density distribution profile, the negative and positive charges are respectively gathered at one side of the formed CoNiMn-C heterointerface, which is beneficial to the formation of “macroscopic” dipole moment and built-in electric field (Fig. 5m). When high-frequency EM waves act, the motion of charges can generate interfacial polarization to absorb the incident EM energy [50–52]. The Cole–Cole curve indicates the existence of multiple relaxation, which may come from interfacial polarization, dipole polarization and defects (Fig. S8) [53]. Simultaneously, the electrons can migrate in the crystal structures and cross the interface energy barrier forming the directional migration routes. Conductive loss in the CoNiMn@C composite can convert the EM energy into thermal energy to realize energy absorption [54]. In addition, lattice distortion in the CoNiMn crystal provides the dipole polarization sites [55]. Furthermore, the geometric phase analysis (GPA) technology is used to observe the certain microstrain distribution, reflecting the lattice changes. Using the HETEM images of CoNiMn@C (Fig. 5h), reconstructed mapping of *E*_*xx*_ and *E*_*yy*_ directional strain diagram are obtained (Fig. 5i, j). The crystal positions corresponding to these color mutation regions exhibit lattice changes, providing the dipole polarization sites. Consequently, tuning ternary magnetic core can enhance the interfacial polarization and conductive loss in the CoNiMn@C composite, realizing efficient EM wave energy dissipation.
(iii) **Constructed hierarchical magnetic coupling in flower-like CoNiMn@C microspheres to boost magnetic attenuation capacity**. EM waves are composed of electric and magnetic fields oscillating in the same phase and are perpendicular to each other. Therefore, the magnetic loss mechanism also plays the key role in EM wave absorption, which is highly relevant to magnetic properties [56]. As shown in Fig. 6a, b, the complex permeability (*μ', μ"*) curves show typical frequency-dependent feature and a decrease tendency. Among those MOFs-derived CoNiM@C composites, the CoNiFe@C composites possess the highest complex permeability because the induced Fe element belongs to the ferromagnetic elements of Group VIII elements. Meanwhile, MOFs-derived CoNiMn@C composites also exhibit high permeability (*μ'*, *μ"*) values, suggesting the strong magnetic loss ability. From the hysteresis loop, the saturation magnetization (*M*_*s*_) and coercive force (*H*_*c*_) can be calculated (Fig. 6c). The *M*_*s*_ values are 78.2 emu g^−1^ for the CoNiCu@C, 32.5 emu g^−1^ for the CoNiZn@C, 18.6 emu g^−1^ for the CoNiMn@C, and 130.8 emu g^−1^ for the CoNiFe@C. According to the formulas of $\mu_{i} = \left( {M_{s}^{2} } \right)/\left( {akH_{c} M_{s} + b\lambda \xi } \right)$, the intrinsic permeability (*µ*_*i*_) is proportional to the *M*_*s*_ and inversely proportional to its coercivity (*H*_*c*_). In other words, the high *M*_*s*_ values are benefited to the improvement of the complex permeability (*µ'*, *µ"*). In the CoNiM@C microspheres system, even though CoNiCu@C and CoNiFe@C hold high *M*_*s*_ values, the wide-frequency absorption performance is not as desired, indicating the importance of balance between the complex permeability and complex permittivity [57]. In addition, the frequency dependence of magnetic loss tangent (tan *δ*_*m*_) is shown in Fig. S9. In other words, a single strong magnetic characteristic is hard to satisfy for broadband absorption in the MOFs-derived CoNiM@C composites.

**Fig. 6:**
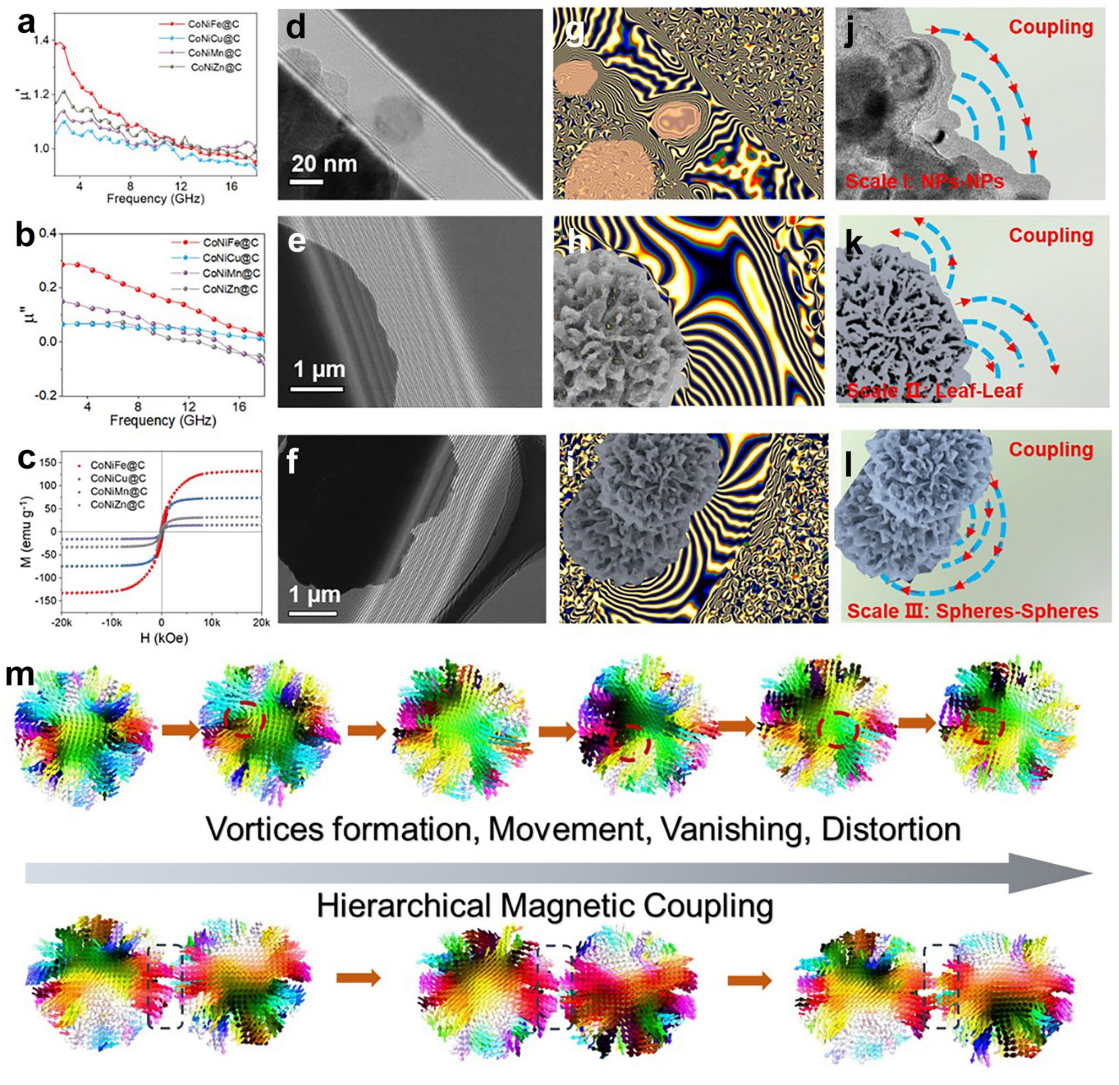
**a** Real and **b** imaginary part of permeability, **c** room-temperature hysteresis loops of CoNiM@C. **d–f** Holography images, **g–i** reconstructed magnetic flux lines distribution and **j–l** diagrams of magnetic flux lines for CoNiMn@C. **m** Computational micro-magnetic simulation of two adjacent CoNiMn@C microspheres

Magnetic loss mainly come from the eddy current loss, resonance natural and exchange resonance in the 2–18 GHz measured frequency [58, 59]. From the changed eddy current loss coefficient *C*_0_ (Fig. S10), those changed data suggesting that magnetic resonance contributes to the magnetic loss rather than the current loss. To study the intrinsic magnetic properties and responding behaviors, electron holography technology was used to observe the magnetic flux line distribution of MOFs-derived CoNiMn@C microsphere at different scale ranges. Based on those holography images (Fig. 6d–f) and phase information, flower-like CoNiMn@C composite can release magnetic field lines at different nano–microscale, exceeding the space volume of the material itself (Fig. 6g–i). At the same time, a strong magnetic coupling phenomenon can be observed. In the flower-like CoNiMn@C composite, hierarchical magnetic coupling can be constructed from nanoscale to micrometer scale. Clearly, magnetic coupling exists among the magnetic nanoparticles–nanoparticles, sheet–sheet, and microsphere–microsphere (Fig. 6j–l). Absolutely, hierarchical magnetic coupling will expand the magnetic responding range, which further promote magnetic loss ability [58, 60]. As the main magnetic loss mechanism, natural resonance and ferromagnetic resonance can consume EM energy by magnetic moment motion [60, 61]. To explore the magnetic configuration and magnetic loss behaviors, micro-magnetic simulation was applied under an alternating magnetic field (Fig. 6m). Different from the smooth microsphere and sheet composites (Figs. S11 and S12), MOFs-derived flower-like CoNiMn@C microsphere shows remarkably morphology change induced by the motion of magnetic moment, including vortices formation, movement, vanishing, and distortion (Figs. 6m and S13). Simultaneously, hierarchical magnetic coupling is also found in the simulation process of applied magnetic field, improving the magnetic responding intensity and ability. Under alternating magnetic field, those motion will absorb the incident EM wave, obstructing further propagation of EM waves.

**Fig. 7:**
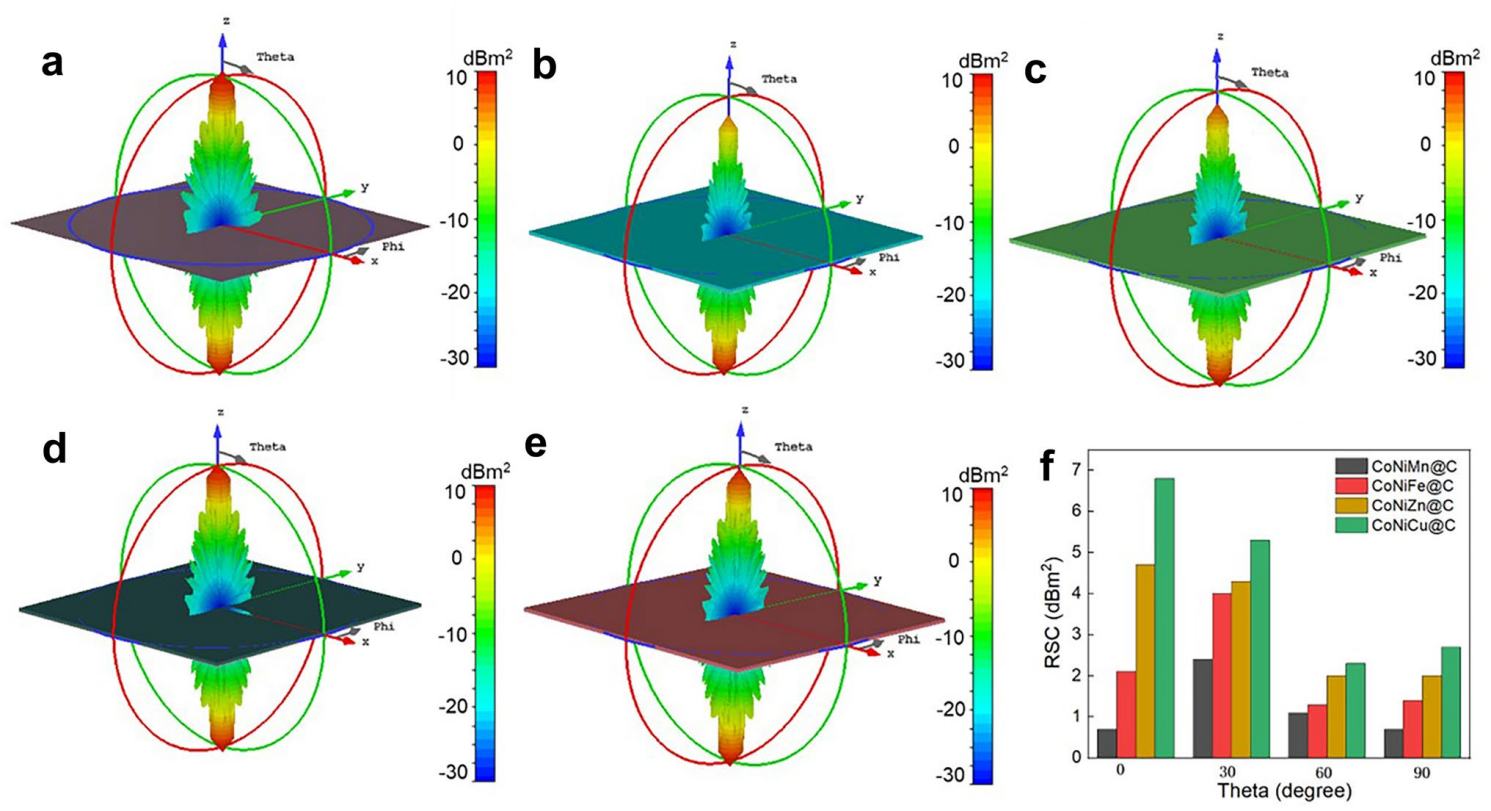
CST simulation results. 3D RCS plots for** a** PEC substrate, PEC substrate covered with **b** CoNiMn@C, **c** CoNiFe@C, **d** CoNiZn@C, and **e** CoNiCu@C, size: 90 × 90 × 2.5 mm^3^. **f** Simulated RCS values of samples under certain detecting angles

To further evaluate the practical application potential, the CST simulation of CoNiM@C microspheres is used to analyze the RCS of a rectangular ultrathin perfect electric conductor (PEC) plate substrate with the size of 90 × 90 × 0.5 mm^3^ (simulated thickness is 2.0 mm). The PEC covered by four CoNiMn@C, CoNiFe@C, CoNiZn@C, and CoNiCu@C composites reveals similar signals of 3D radar wave (Fig. 7a–e). Apparently, weaker RCS signals are obtained after coating CoNiM@C than pure PEC without any coating layer. The radiation lobe structure and color change of CoNiMn@C coating show the weakest reflected signal, demonstrating most EM energy is propagated (Fig. 7b). Figure 7f depicts the RCS reduction effect at different detection angles at 14.2 GHz in the Ku band (0°, 30°, 60°, 90°, subtracting the RCS value of the PEC substrate). Hence, flower-like CoNiMn@C is superior to that of other CoNiM@C samples, and the corresponding RCS values are 0.7, 2.4, 1.7, and 0.1 dB m^2^ at 0°, 30°, 60°, and 90°, respectively, elucidating the maximum EM wave absorption capacity at different detection angles (Fig. 7f). This simulated result is consistent well with the excellent EM wave absorption capacity in Fig. 3.

## Conclusion

In summary, MOFs-derived magnetic-carbon CoNiM@C (M = Cu, Zn, Fe, Mn) microspheres assembled by sheets are designed and fabricated. After introducing the adjacent elements into magnetic CoNi alloy, MOFs-derived CoNiM@C microspheres displayed evolutionary flower-like morphology. The CoNiM@C composites possess controllable nano–microstructure to regulate EM parameters. Among those CoNiM@C microspheres, CoNiMn@C holds the outstanding broadband absorption and the EAB of 5.8 GHz at 2.0 mm thickness, covering from 12.2 to18 GHz. And the order of efficient EAB value is Mn > Fe = Zn > Cu in the series of CoNiM@C microspheres. To explore the inherent dielectric dissipation and magnetic loss, unique off-axis electron holography, micro-magnetic simulation and CST simulation are used. It was found that the magnetic alloy–carbon heterointerface can enhance the gathering of charges at the contacting region, which is necessary and further contribute to the interfacial polarization. The graphitized carbon layer offered conductive loss accompanied by the directional migration of electrons. Finally, hierarchical magnetic coupling is visually observed in the CoNiMn@C microspheres, which strengthened the magnetic responding and absorption ability. Combined MOFs-derived strategy and magnetic alloys regulation provide a new path to design and build broadband EM absorption materials.

## Supplementary Information

Below is the link to the electronic supplementary material.

Supplementary file1 (DOCX 5174 KB)
