# Supplementary material for: MOFs-Derived Strategy and Ternary Alloys Regulation in Flower-Like Magnetic-Carbon Microspheres with Broadband Electromagnetic Wave Absorption
Source: Nanomicro Lett. 2024 Jul 12;16:245. doi: 10.1007/s40820-024-01416-2 (PMC11245463; doi:10.1007/s40820-024-01416-2)
Supplement: Supplementary file 1 — Supplementary file1 (DOCX 5174 KB) [file 40820_2024_1416_MOESM1_ESM.docx]

Supporting Information for

**MOFs Derived Strategy and Ternary Alloys Regulation in Flower-Like Magnetic-Carbon Microspheres with Broadband Electromagnetic Wave Absorption**

Mengqiu Huang^1,#^, Bangxin Li^2,#^, Yuetong Qian^3,#^, Lei Wang^4^, Huibin Zhang^3^, Chendi Yang^1^, Longjun Rao^1^, Gang Zhou^1^, Chongyun Liang^2,^* and Renchao Che^1,5,6,^*

^1^Laboratory of Advanced Materials, Shanghai Key Lab of Molecular Catalysis and Innovative Materials, Academy for Engineering & Technology, Fudan University, Shanghai 200438, P. R. China

^2^Department of Chemistry, Fudan University, Shanghai 200438, P. R. China

^3^Materials Genome Institute, Shanghai University, Shanghai 200444, P. R. China

^4^School of Materials Science and Engineering, Shanghai Institute of Technology, Shanghai 201418, P. R. China

^5^College of Physics, Donghua University, Shanghai 201620, P. R. China

^6^Zhejiang Laboratory, Hangzhou 311100, P. R. China

^#^Mengqiu Huang, Bangxin Li, and Yuetong Qian contributed equally to this work.

*Corresponding authors. E-mail: [cyliang@fudan.edu.cn](mailto:cyliang@fudan.edu.cn) (Chongyun Liang); [rcche@fudan.edu.cn](mailto:rcche@fudan.edu.cn) (Renchao Che)

**Supplementary Figures**


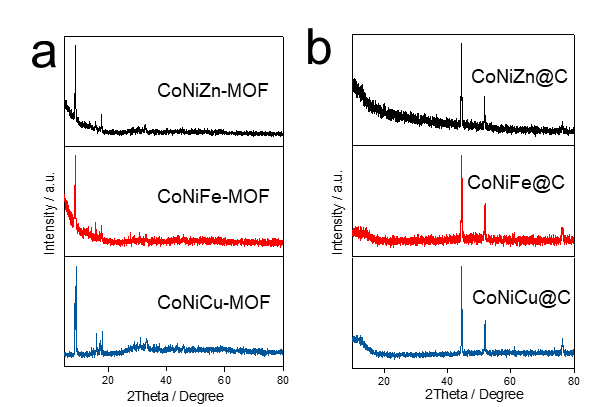


**Fig. S1** XRD patterns of **a** CoNiZn-MOF, CoNiFe-MOF, CoNiCu-MOF precursors and **b** CoNiZn@C, CoNiFe@C, CoNiCu@C composites


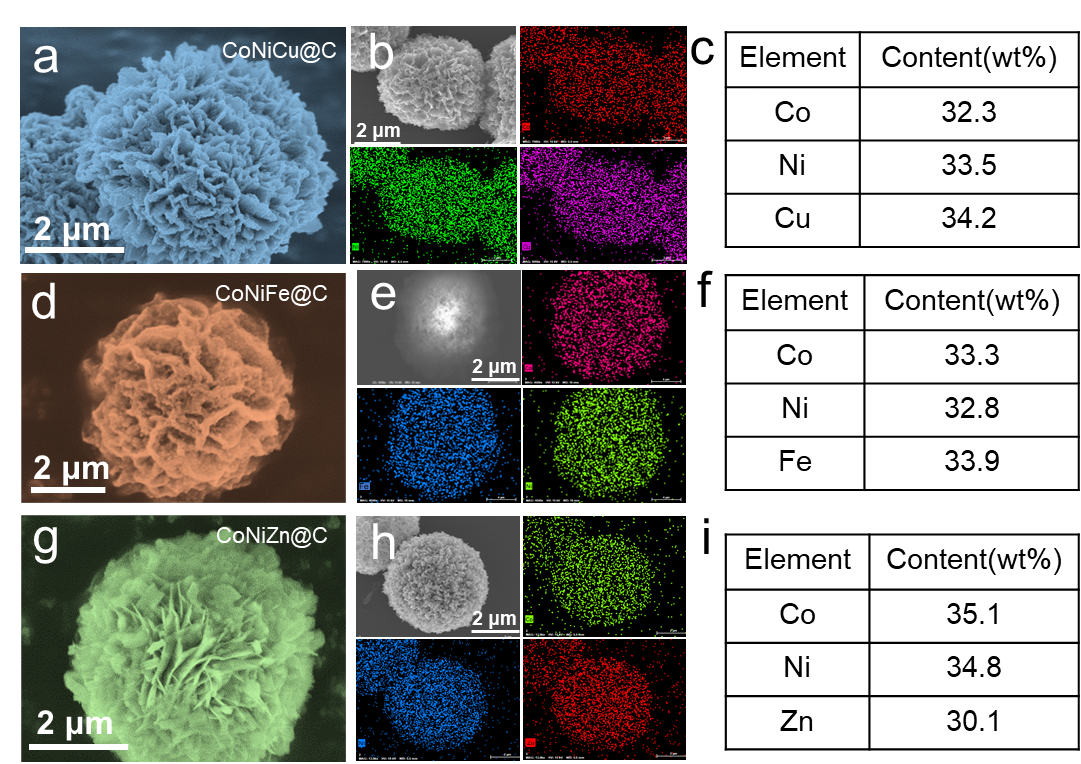


**Fig. S2** SEM images and EDX mapping of **a-c** CoNiCu@C, **d-f** CoNiFe@C, and **g-i** CoNiZn@C


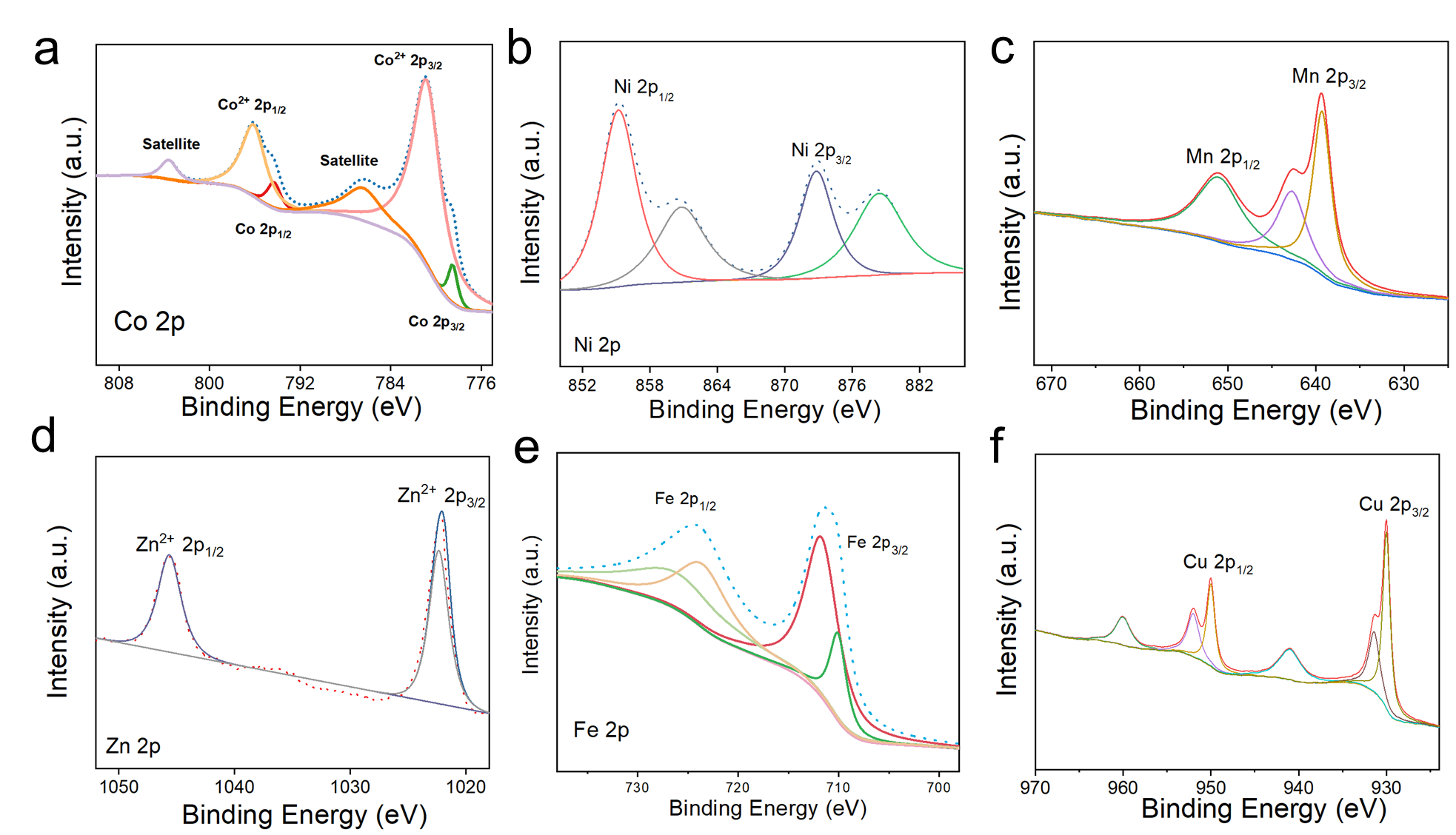


**Fig. S3** High resolution XPS spectra of **a** Co 2p, **b** Ni 2p, **c** Mn 2p, **d** Zn 2p, **e** Fe 2p and **f** Cu 2p for CoNiM@C microsphere


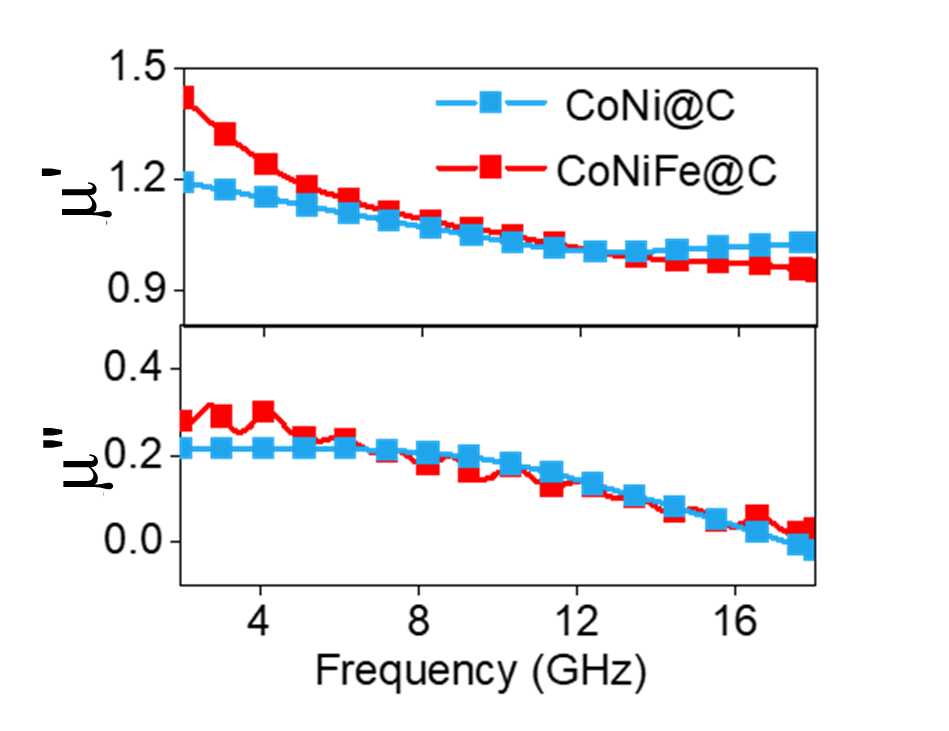


**Fig. S4** Real and imaginary part of permeability of CoNi@C and CoNiFe@C

**Fig. S5** Room-temperature hysteresis loops of CoNi@C and CoNiFe@C


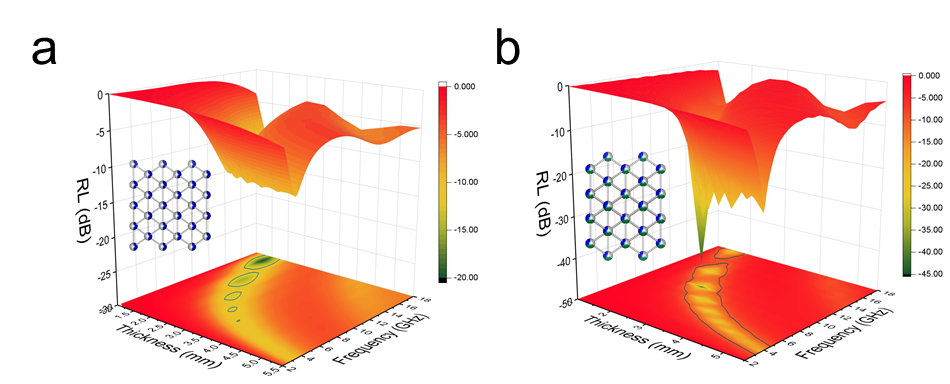


**Fig. S6** 3D reflection loss mapping of **a** CoNi@C and **b** CoNiFe@C


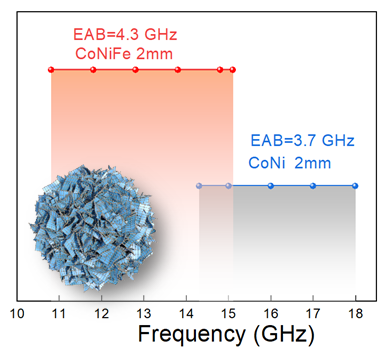


**Fig. S7** Effective absorption bandwidth of CoNi@C and CoNiFe@C


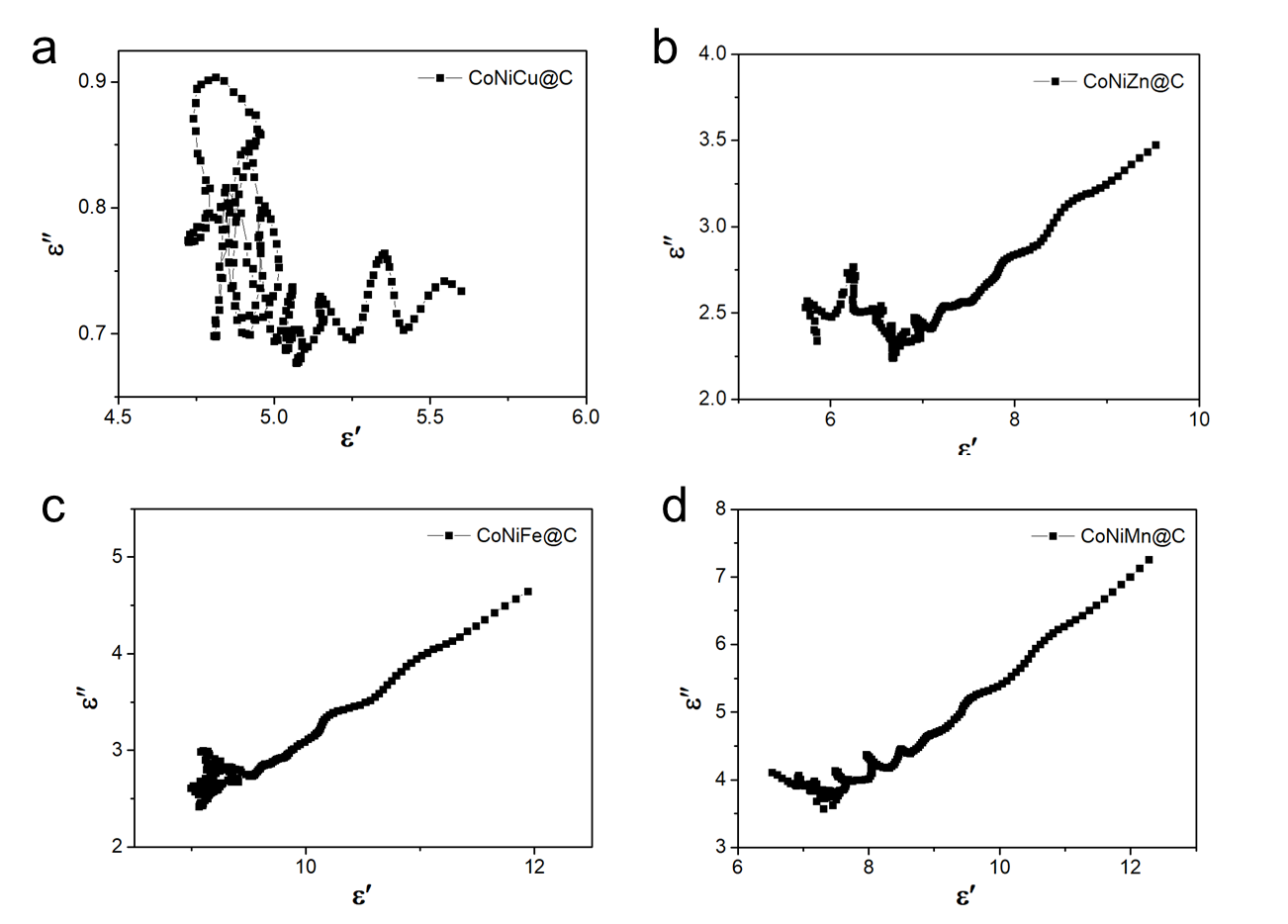


**Fig. S8** Cole-Cole semicircular of CoNiM@C microspheres


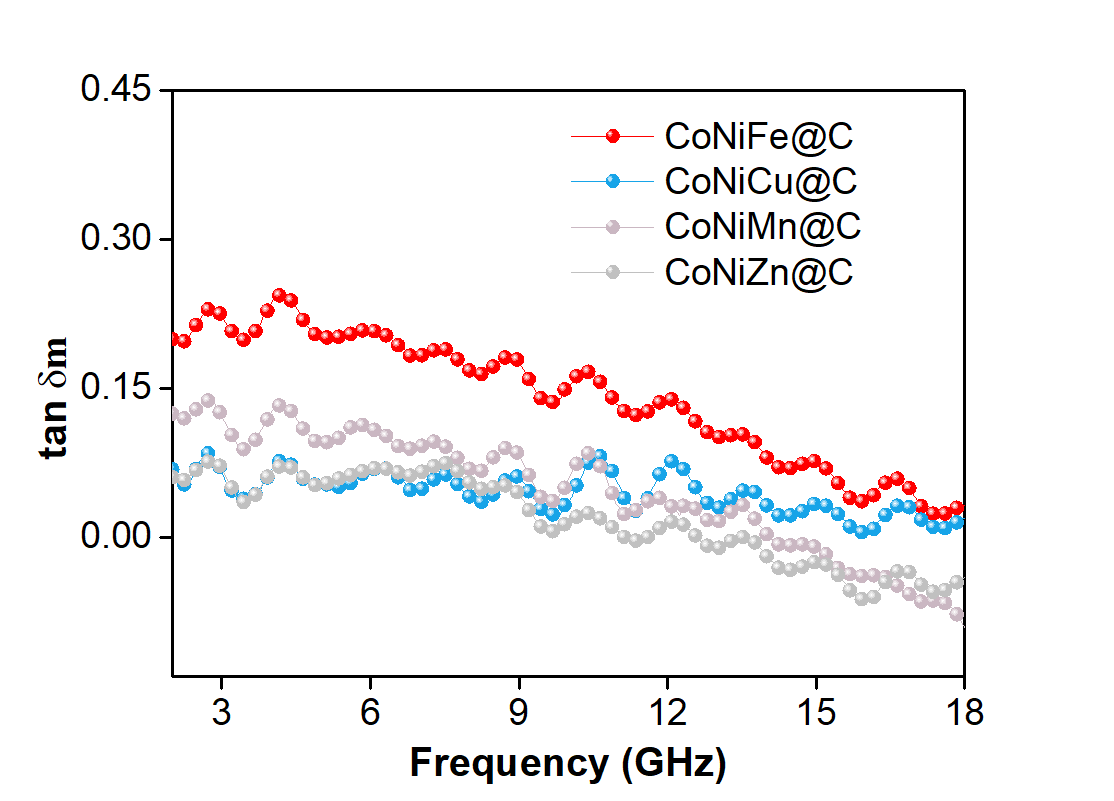


**Fig. S9** The magnetic loss tangent value of CoNiM composites


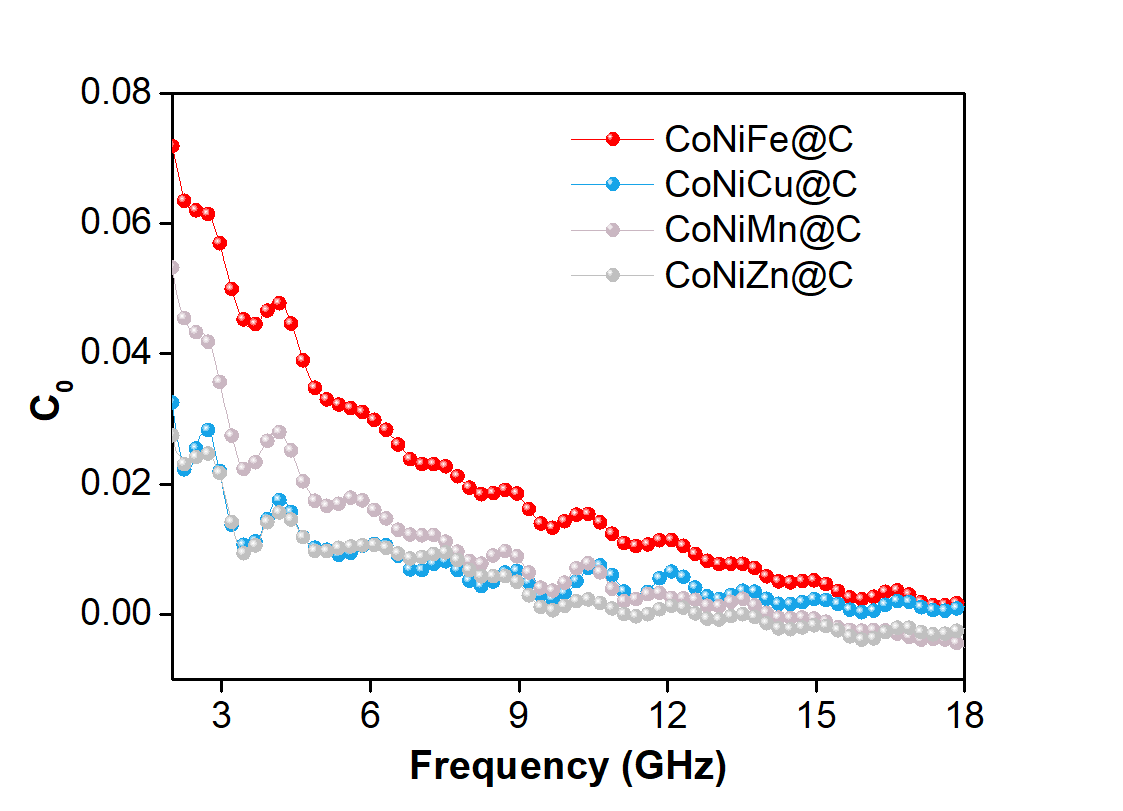


**Fig. S10** The eddy current loss coefficient C_0_ value of CoNiM composites


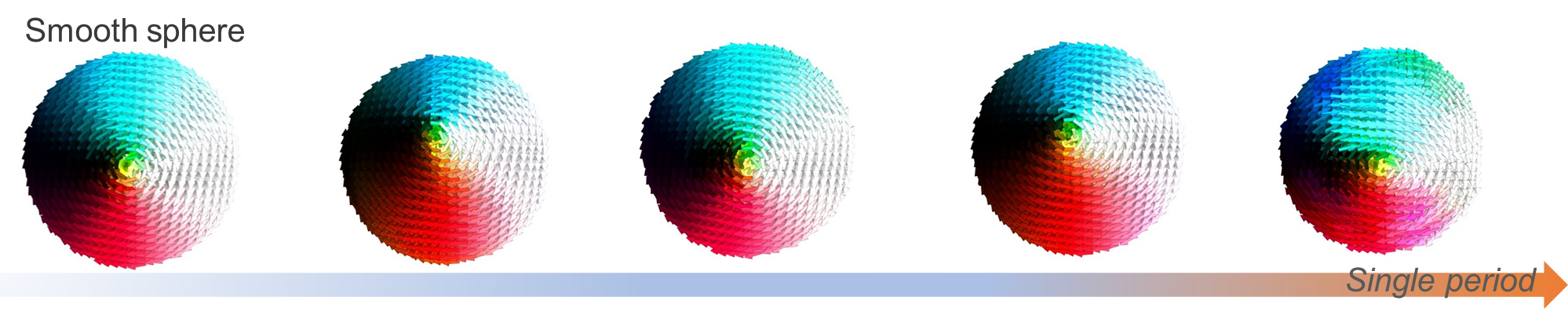


**Fig. S11** The simulated magnetic moments in smooth microspheres


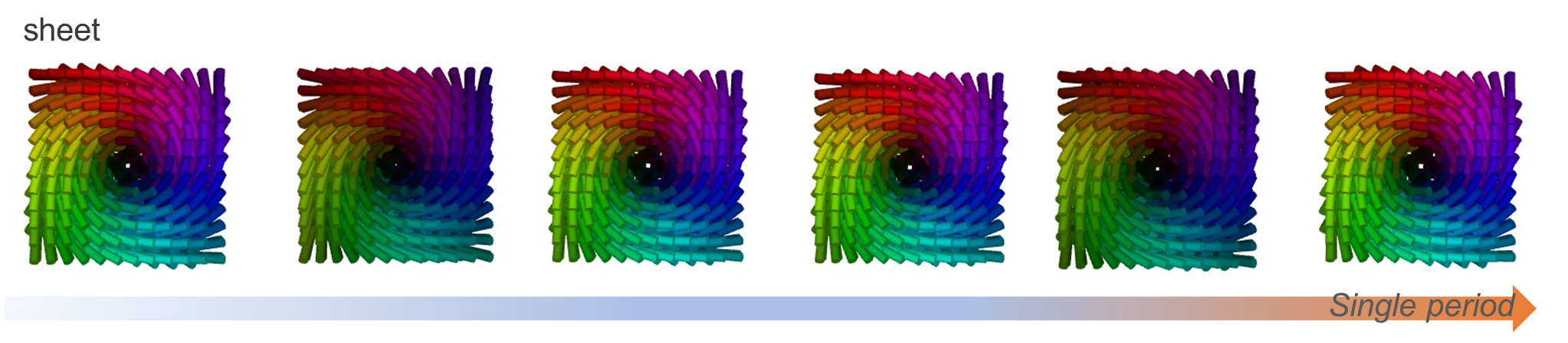


**Fig. S12** The simulated magnetic moments in sheets


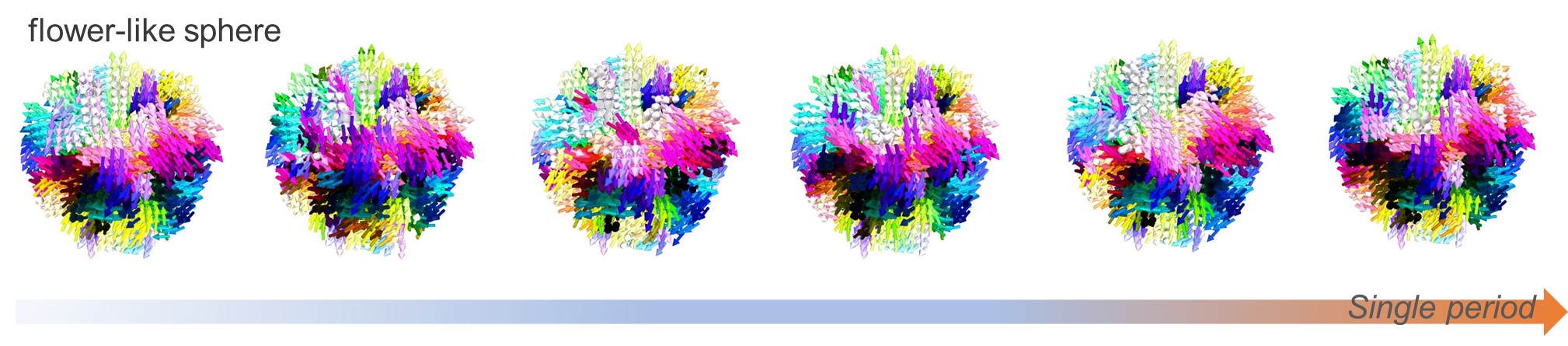


**Fig. S13** The simulated magnetic moments in flower-like microspheres

**Supplementary Table**

**Table S1** The electromagnetic wave absorption performance comparison of CoNi-based composites

| Sample | Filler loading (wt %) | Thickness  (mm) | EAB (GHz) | Refs. |
| --- | --- | --- | --- | --- |
| CoNi microspheres | 60 | 1 | 2.96 | [S1] |
| CoNi chains | 60 | 5.4 | 3.5 | [S2] |
| CoNi Nanoparticles | 65 | 1.4 | 4.4 | [S3] |
| CoNi@NC hollow microspheres | 30 | 3.08 | 4.5 | [S4] |
| CoNiMPC @CNT/MXene | 15 | 2.7 | 5.0 | [S5] |
| CoNi@graphite carbon@carbon | 70 | 2.0 | 5.51 | [S6] |
| CoNi/C hybrids | 20 | 2.1 | 5.7 | [S7] |
| Co_x_Ni_y_@C nanocomposites | 20 | 1.8 | 5.7 | [S8] |
| CoNi@NC/rGO-600 | 25 | 2.5 | 6.7 | [S9] |
| CoNi microflowers | 16.7 | 2 | 6.8 | [S10] |
| **CoNiMn@C flower** | **40** | **2** | **5.8** | **This work** |

EAB: Efficient absorption bandwidth

**Supplementary References**

1. Z. Wang, W. Yang, Q. Lv, S. Liu, Z. Fang, Ferromagnetic and excellent microwave absorbing properties of CoNi microspheres and heterogeneous Co/Ni nanocrystallines. RSC Adv. **9**(24), 13365-13371 (2019). https://doi.org/ 10.1039/C9RA02013F
2. M. Qiao, J. Li, S. Li, D. Wei, X. Lei, Hierarchical CoNi alloys toward microwave absorption application: Chain-like versus particle-like. J. Alloy Compd. **926**, 166854 (2022). <https://doi.org/10.1016/j.jallcom.2022.166854>
3. J. Park, D. Ahn, J. Ro, S. Suh, CoNi Nanoparticles with different compositions using a polyol method for a microwave absorber in high-frequency bands. Met. Mater. Int. **29**(5), 1542-1554 (2023). <https://doi.org/10.1007/s12540-022-01298-2>
4. W. Min, D.Xu, P. Chen, G. Chen, Q. Yu et al., Synthesis of novel hierarchical CoNi@NC hollow microspheres with enhanced microwave absorption performance. J. Mater. Sci-Mater El. **32**, 8000-8016 (2021). <https://doi.org/10.1007/s10854-021-05523-3>
5. T. Wu, F. Ren, Z. Guo, J. Zhang, X. Hou et al., Bayberry-like bimetallic CoNi-MOF-74 derivatives/MXene hybrids with abundant heterointerfaces toward high-efficiency electromagnetic wave absorption. J. Alloy. Compd. **976**, 172984 (2024). <https://doi.org/10.1016/j.jallcom.2023.172984>
6. X. Wu, W. Ma, J. Xu, P. He, Y. Du, et al., Hierarchical Multi-Core–Shell CoNi@Graphite Carbon@Carbon Nanoboxes for Highly Efficient Broadband Microwave Absorption. ACS Appl. Nano Mater. **5**(5), 7300-7311 (2022). <https://doi.org/10.1021/acsanm.2c01215>
7. J. Ge, Y. Cui, J. Qian, L. Liu, F. Meng et al., Morphology-controlled CoNi/C hybrids with bifunctions of efficient anti-corrosion and microwave absorption. J. Mater. Sci. Technol. **102**, 24-35 (2022). <https://doi.org/10.1016/j.jmst.2021.07.003>
8. X. Liang, Z. Man, B. Quan, J. Zheng, W. Gu et al., Environment-stable Co_x_Ni_y_ encapsulation in stacked porous carbon nanosheets for enhanced microwave absorption. Nano-Micro Lett. **12**, 102 (2020). <https://doi.org/10.1007/s40820-020-00432-2>
9. X. Xu, F. Ran, Z. Fan, Z. Cheng, T. Lv et al., Bimetallic metal-organic framework-derived pomegranate-like nanoclusters coupled with CoNi-doped graphene for strong wideband microwave absorption. ACS Appl. Mater. Interfaces **12**(15), 17870-17880 (2020). <https://doi.org/10.1021/acsami.0c01572>
10. Q. Liu, Qi Cao, X. Zhao, H. Bi, C. Wang, Insights into size-dominant magnetic microwave absorption properties of CoNi microflowers via off-axis electron holography. ACS Appl. Mater. Interfaces **7**(7), 4233-4240 (2015) <https://doi.org/10.1021/am508527s>
